# Supplementary material for: Measurement Invariance of the WHO-5 Well-Being Index: Evidence from 15 European Countries
Source: Int J Environ Res Public Health. 2022 Aug 9;19(16):9798. doi: 10.3390/ijerph19169798 (PMC9407912; doi:10.3390/ijerph19169798)
Supplement: Supplementary file 1 [file ijerph-19-09798-s001.zip › ijerph-1857435-supplementary.pdf]

Supplementary Materials:

Table S1. WHO-5 Well-being Index, Table CFA model fit and reliability, by country (*N* = 79,104, 15 countries)

Country	*n*	CFI	TLI	RMSEA	SRMR	Min. loading	Max. loading	Internal consistency
Overall	79,104	0.983	0.965	0.084	0.020	0.70	0.77	0.860
Austria	4,129	0.977	0.065	0.091	0.023	0.64	0.76	0.835
Armenia	4,717	0.988	0.971	0.058	0.017	0.60	0.73	0.800
Czechia	11,564	0.963	0.925	0.165	0.028	0.76	0.89	0.918
Georgia	4,242	0.968	0.935	0.142	0.029	0.73	0.88	0.903
Ireland	3,833	0.973	0.946	0.114	0.024	0.70	0.82	0.872
Kazakhstan	4,868	0.991	0.984	0.041	0.013	0.50	0.72	0.752
Lithuania	3,797	0.982	0.963	0.081	0.020	0.68	0.77	0.835
Republic of Moldova	4,686	0.981	0.963	0.069	0.021	0.60	0.70	0.785
Poland	5,224	0.979	0.958	0.081	0.021	0.65	0.75	0.816
Romania	4,567	0.979	0.959	0.080	0.022	0.63	0.73	0.812
Russian Federation	4,281	0.976	0.952	0.096	0.023	0.66	0.80	0.844
Scotland	5,021	0.985	0.970	0.080	0.017	0.71	0.79	0.861
Slovenia	5,667	0.980	0.961	0.092	0.022	0.63	0.82	0.854
Turkey	5,848	0.967	0.935	0.105	0.026	0.53	0.77	0.817
Ukraine	6,660	0.981	0.963	0.104	0.020	0.74	0.86	0.898

CFA = confirmatory factor analysis; CFI = comparative fit index; TLI = Tucker Lewis Index;
RMSEA = root mean square error of approximation; SRMS = standardised root mean square residual

Table S2. WHO-4 Well-being Index, Table CFA model fit and reliability, by country (*N* = 74,071, 15 countries)

Country	*n*	CFI	TLI	RMSEA	SRMR	Min. loading	Max. loading	Internal consistency
Overall	74,071	0.998	0.993	0.041	0.009	0.71	0.76	0.860
Austria	3,988	0.996	0.988	0.048	0.011	0.67	0.73	0.799
Armenia	4,374	0.991	0.971	0.066	0.017	0.59	0.71	0.752
Czechia	9,813	0.994	0.983	0.083	0.012	0.81	0.86	0.897
Georgia	3,856	0.967	0.902	0.190	0.033	0.75	0.89	0.881
Ireland	3,636	0.999	0.997	0.026	0.006	0.70	0.78	0.838
Kazakhstan	4,354	0.998	0.994	0.025	0.008	0.52	0.68	0.701
Lithuania	3,710	0.998	0.995	0.030	0.008	0.68	0.71	0.796
Republic of Moldova	4,476	0.994	0.981	0.052	0.014	0.63	0.68	0.743
Poland	5,127	0.999	0.996	0.027	0.007	0.66	0.72	0.777
Romania	4,392	0.995	0.986	0.048	0.012	0.63	0.73	0.774
Russian Federation	4,104	1.000	0.999	0.014	0.005	0.63	0.80	0.805
Scotland	4,852	0.998	0.995	0.035	0.008	0.71	0.74	0.826
Slovenia	5,540	0.997	0.992	0.044	0.010	0.66	0.78	0.818
Turkey	5,671	0.998	0.994	0.034	0.009	0.54	0.78	0.774
Ukraine	6,178	0.995	0.985	0.069	0.012	0.77	0.84	0.870

CFA = confirmatory factor analysis; CFI = comparative fit index; TLI = Tucker Lewis Index;
RMSEA = root mean square error of approximation; SRMS = standardised root mean square residual

Table S3. Convergent validity: correlation coefficients using aggregated data (WHO-5) (N = 74,071)

	1	2	3	4
1. WHO-5 Well-being	1			
2. Psychosomatic complaints	−0.455	1		
3. Life satisfaction	0.446	−0. 376	1	
4. Self-rated health	−0.341	0.334	−0.371	1

All correlations are significant at $p < 0.05$.

Table S4. Convergent validity by gender: correlation coefficients using aggregated data (WHO-5) (N = 74,071)

	1 (boys)	2 (boys)	3 (boys)	4 (boys)
1. WHO-5 Well-being (girls)	1	−0.383	0.398	−0.298
2. Psychosomatic complaints (girls)	−0.496	1	-0.319	0.276
3. Life satisfaction (girls)	0.483	−0.417	1	−0.349
4. Self-rated health (girls)	−0.369	0.382	-0.386	1
